# The effect of *Nigella Sativa* emulgel on episiotomy wound healing and pain intensity in primiparous women: A triple-blind randomized controlled trial

**DOI:** 10.1371/journal.pone.0325112

**Published:** 2025-06-04

**Authors:** Mahsa Maghalian, Afsaneh Alizadeh, Fatemeh Raphi, Ziba Islambulchilar, Laleh Khodaie, Mahsan Nabighadim, Simin Taghavi, Mojgan Mirghafourvand

**Affiliations:** 1 Student Research Committee, Tabriz University of Medical Sciences, Tabriz, Iran; 2 Department of Midwifery, Faculty of Nursing and Midwifery, Tabriz University of Medical Sciences, Tabriz, Iran; 3 Master of Midwifery, Clinical Research Development Unit, Taleghani Hospital, Tabriz University of Medical Sciences, Tabriz, Iran; 4 Department of Pharmaceutics, Faculty of Pharmacy, Tabriz University of Medical Sciences, Tabriz, Iran; 5 Department of Pharmacognosy, Faculty of Pharmacy, Tabriz University of Medical Sciences, Tabriz, Iran; 6 Medicine student, Medical School, Ardabil University of Medical Sciences, Ardabil, Iran; 7 Obstetrics and Gynecology, Women’s Reproductive Health Research Center, Tabriz University of Medical Sciences, Tabriz, Iran; 8 Social Determinants of Health Research Center, Tabriz University of Medical Sciences, Tabriz, Iran; University of Nairobi, KENYA

## Abstract

**Background:**

Episiotomy, a common surgical procedure during childbirth, often leads to complications such as pain, infection, and delayed healing. *Nigella sativa* has demonstrated anti-inflammatory and wound-healing properties in prior studies and have received United States Food and Drug Administration (FDA) approval for food use, indicating their safety. This study aimed to evaluate the efficacy of *Nigella sativa* emulgel on episiotomy wound healing and pain intensity in primiparous women.

**Methods:**

A triple-blind, randomized controlled trial was conducted at Taleghani Hospital, Tabriz, Iran (May 2023–April 2024). Seventy-four primiparous women with mediolateral episiotomy were randomized to receive either *Nigella sativa* emulgel or placebo, applied topically three times daily for 7 days post-discharge. Wound healing was assessed using the REEDA scale (Redness, Edema, Ecchymosis, Discharge, Approximation; primary outcome), and pain intensity was measured via visual analog scale (VAS; secondary outcome). Outcomes were evaluated at discharge (baseline) and 10 ± 1 days postpartum. Data were analyzed using independent t-tests, ANCOVA (adjusted for baseline scores), and Mann-Whitney U tests for non-normal distributions (SPSS v26).

**Results:**

At 10 ± 1 days postpartum, the *Nigella sativa* group showed significantly better wound healing (REEDA score: MD −0.79, 95% CI −1.20 to −0.39; p = 0.001) and lower pain scores (VAS: MD −0.74, 95% CI −1.3 to −0.11; p = 0.021) compared to placebo. Subscale analysis revealed improvements in redness (p = 0.037), edema (p = 0.041), and ecchymosis (p = 0.043). No adverse effects were reported, and satisfaction was higher in the *Nigella sativa* group (86.5% vs. 56.7%; p = 0.046).

**Conclusions:**

Topical *Nigella sativa* emulgel significantly improved episiotomy wound healing and reduced pain intensity, with high patient satisfaction. These findings support its potential as a natural therapeutic option, though larger multi-center trials are needed for broader validation.

**Trial registration:**

Iranian Registry of Clinical Trials IRCT20120718010324N68.

## Introduction

Episiotomy is a surgical procedure performed during childbirth that involves making an incision in the perineal muscles. The goal is to widen the soft tissue of the pelvic outlet and reduce the risk of severe tearing in obstetrics [1,2].

The World Health Organization (WHO) and International Federation of Gynecology and Obstetrics (FIGO) have recommended that healthcare policies should restrict the use of episiotomy to no more than 10% of birth [3–5]. Episiotomy rates vary globally, with the lowest in Sweden (9.7%) and the highest in Taiwan (100%) [6]. Asian women often undergo episiotomy due to their shorter perineum and higher risk of tearing [7–9]. Studies conducted in Iran have reported episiotomy rates of 41.5% (50.8% in primiparous vs. 40.16% in multiparous women) and 54.5% (87% in primiparous vs. 41.8% in multiparous women), significantly exceeding the globally recommended maximum rate of 10% [10,11]. Episiotomy is linked to increased perineal pain that can last for over two weeks and up to five months postpartum [12–15]. Perineal pain from episiotomy can negatively impact the mother’s quality of life, healing, bonding with the newborn, self-care, and increase the risk of postpartum depression [12,16,17]. Episiotomy is further associated with long-term adverse effects, such as sexual dysfunction and diminished sexual satisfaction, underscoring the need for effective interventions [18].

Various pharmacological options are available to manage episiotomy-related pain, including acetaminophen, non-steroidal anti-inflammatory drugs (NSAIDs), intravenous (IV) opioids, and topical anesthetics. However, these interventions can have side effects such as excretion in breast milk, especially in the early postpartum period, and in the case of intravenous opioids, they can lead to itching, nausea, and respiratory depression [19,20].

In addition to pharmacological interventions, various non-pharmacological treatments can be used to manage episiotomy-related pain, such as cold packs or chemical coolants, laser therapy, electrical stimulation, acupuncture, and pelvic floor exercises [21,22]. Furthermore, education and increasing awareness about perineal wound care can also be an effective non-pharmacological intervention [21]. Herbal medicines have also been studied as a low-cost and non-pharmacological approach to reducing pain and promoting episiotomy healing. These include products derived from olive [22], lavender [23], aloe vera [24], and cinnamon [25]. Animal and human studies have shown that *Nigella Sativa* can promote the healing of wounds other than episiotomy wounds without significant side effects [26–28].

Wound healing is a physiological process that occurs in response to tissue injury or damage [29]. *Nigella Sativa*, also known as black cumin, black seeds, black coriander, or black cumin seeds, has been found to have anti-inflammatory, analgesic, and antioxidant properties.

It is considered a safe substance, as it has been approved for food use by the United States Food and Drug Administration (FDA) [30,31]. Black cumin seeds are a nutritional powerhouse, packed with an array of vitamins, minerals, fiber, amino acids, and beneficial compounds like linoleic acid, thymoquinone, and antioxidants. They are an excellent source of high-quality oil and protein, and their health-promoting properties make them a valuable addition to the diet. Black cumin seeds can be utilized both as a nutritional oil and a direct food source to help meet people’s daily dietary needs [32].

Black cumin along with its main component thymoquinone, has been employed in the treatment of various health issues, such as diabetes, infertility, respiratory problems, neurological disorders, cancer, and skin conditions [32,33]. The plant’s oil is readily absorbed through the skin due to its high content of beneficial fatty acids like linoleic acid [34]. A review study examining the effects of *Nigella Sativa* on various skin conditions, such as psoriasis, acne vulgaris, skin pigmentation, hypersensitivity reactions, and skin cancers, has demonstrated the plant’s significant therapeutic potential in dermatology [35].

Given the potential benefits of Nigella Sativa, its low cost, and the growing preference for non-pharmacological approaches [36], investigating the use of *Nigella Sativa* emulgel for episiotomy management could provide a valuable, accessible, and natural alternative to conventional treatments. Therefore, the proposed study aims to fill this research gap by evaluating the efficacy of *Nigella Sativa* emulgel in promoting episiotomy repair and reducing associated pain in postpartum women. The findings could contribute to the development of evidence-based, patient-centered approaches to episiotomy management and postpartum care. We hypothesized that topical application of *Nigella sativa* emulgel would significantly enhance episiotomy wound healing and reduce pain intensity compared to placebo treatment.

## Methods

### Study design and participants

This was a randomized, triple-blind (participants, outcome assessors, and data analysts) controlled clinical trial conducted at the Taleghani Educational-Medical Center in Tabriz, Iran. The study took place from May 19, 2023 to April 15, 2024. The participants included 74 primiparous women who had undergone a mediolateral episiotomy.

### Eligibility criteria

The inclusion criteria for this study are primiparous women with live, singleton births. The exclusion criteria include a history of substance abuse or use of specific medications, underlying conditions that could impact wound healing, special dietary regimens, severe anemia during pregnancy, early postpartum hemorrhage, Operative vaginal delivery (vacuum or forceps assistance), large or extended episiotomy, inability to attend the study site on day 10 postpartum, and prolonged rupture of membranes (greater than 18 hours).

### Randomization and blinding

The treatment allocation sequence was generated using block randomization with variable block sizes of 4 and 6, and a 1:1 allocation ratio between the treatment and control groups. The randomization sequence was created using the random number generator provided by the website random.org. To maintain concealment, identical, opaque, sequentially numbered containers were used. These containers were provided to participants in the order they enrolled in the study. The generation of the treatment allocation sequence and the preparation of the containers were conducted by an individual who was not involved in the participant enrollment, treatment allocation, or data collection processes of the study.

### Interventions and follow-up

After obtaining ethical approval (IR.TBZMED.REC.1400.838) and registering the study in the Iranian Registry of Clinical Trials (IRCT20120718010324N68), the researcher obtained permission for participant sampling from the Research Deputy of the School of Nursing and Midwifery and Tabriz University of Medical Sciences. The researchers then introduced themselves to the director of the Taleghani educational and treatment center in Tabriz and were stationed in the delivery ward. The researcher subsequently approached mothers who had recently given birth and, if they met the eligibility criteria, explained the study objectives and provided the necessary information. Written informed consent was obtained, and the participants completed a personal-social and midwifery questionnaire.

The study hospital provided standardized postpartum care for episiotomy patients, consistent with routine clinical practice. This included comprehensive education on perineal wound management (cleaning with warm water after each voiding, proper drying techniques, and avoidance of contaminating activities), guidance on oral analgesic use for pain control (typically acetaminophen as needed), and instruction to monitor for signs of infection. Notably, the institutional protocol did not prescribe any topical pharmacological treatments for episiotomy wounds, reflecting the current absence of globally standardized interventions for postnatal perineal care [37].

In the 24 hours prior to the intervention, which was the time the patient was admitted to the delivery ward for childbirth, a small amount of *Nigella Sativa* emulgel was applied to the skin of the patient’s arm to check for any potential skin sensitivity. If no sensitivity was observed, the participants were then allowed to use the emulgel on the episiotomy site. The length of the episiotomy incision was measured using a sterile tape. Participants were instructed to apply the *Nigella sativa* emulgel daily to the episiotomy site, without massaging, three times a day for one week following their discharge. A tracking sheet was provided for participants to mark each daily application. Participants were given a contact number for any questions or issues.

On day 10 ± 1 after childbirth, participants were asked to bring the completed intervention checklist and the container of the used intervention product to their visit. They were also instructed to report any adverse events, such as sensitivity or infection, that occurred after using the intervention. If any issues arose, the intervention was stopped and the complications were documented. Additionally, the researcher provided written and verbal training on topics like perineum and suture care, personal hygiene, nutrition, physical activity, and proper use of the *Nigella Sativa* emulgel.

### Preparation of *Nigella Sativa* and placebo emulgels

Emulgel formulations were prepared in two distinct steps: first, the emulsion and gel phases were created separately, and then these two formulations were combined to produce the final emulgel product.

### Emulsion preparation

To prepare the emulsion formulation, the lipophilic emulsifier Span 80 (0.5% w/w) was initially dissolved in the oil phase, which consisted of either black cumin oil (for the *Nigella Sativa* emulgel, or investigation group) or paraffin (for the placebo emulgel, or control group). Concurrently, the hydrophilic emulsifier Tween 80 (0.5% w/w) was dissolved in purified water. The preservatives methyl paraben (0.1% w/w) and propyl paraben (0.01% w/w), along with the humectant propylene glycol (5% w/w), were subsequently added to the aqueous phase. Both the oil and aqueous phases were heated to a temperature range of 70–80°C. The oil phase was then gradually incorporated into the aqueous phase while continuously stirring, allowing the resulting emulsion to cool to room temperature.

### Gel component preparation

For the gel component, carbomer 940 (2% w/w) was dispersed in purified water and subjected to agitation at 700 rpm. A small quantity of triethanolamine was added, which raised the pH and facilitated the swelling of the carbomer, resulting in the formation of a viscous gel. This gel base was then mixed with the previously prepared emulsion in a 1:1 ratio to create the final emulgel formulation.

### Sterilization process

Given that the complete formulation cannot undergo terminal sterilization under typical laboratory conditions, the individual components were sterilized separately. The aqueous phase was autoclaved, while the oily phase was sterilized using an oven. Additionally, all necessary manufacturing equipment and packaging containers were sterilized prior to use.

### Data collection tools

Sociodemographic Data Collection: Episiotomy-related outcomes included incision length (measured post-delivery using a sterile ruler) and the number of skin sutures required. Body mass index (BMI) was calculated based on weight and height measurements obtained from first-trimester medical records. Additional clinical variables included the duration of the second and third stages of labor (recorded in minutes). Neonatal birth weight was assessed using a calibrated digital scale.

Sociodemographic variables comprised educational attainment (categorized as primary, secondary, high school, diploma, or university), employment status (for both participants and their spouses), and self-reported household income adequacy (classified as completely sufficient, relatively sufficient, or insufficient). Further covariates included the childbirth attendant (midwife, resident physician, or midwifery student supervised by an instructor) and postpartum toilet type (squat or sitting).

The questionnaire’s content validity was assessed through expert review by ten midwifery and reproductive health specialists from Tabriz University of Medical Sciences. Face validity was subsequently evaluated via cognitive interviews with ten postpartum women to ensure item clarity.

Episiotomy and Childbirth Characteristics Questionnaire: This questionnaire included the length of the episiotomy incision, the number of sutures at the episiotomy site, the duration of the first, second, and third stages of labor, the mode of delivery, and the type of sanitary pads used after childbirth.

Visual Analog Scale (VAS): The VAS questionnaire was used to measure the participants’ pain intensity. The VAS consists of a 10-centimeter graduated ruler, with the numbers ranging from 0 (indicating no pain) to 10 (representing the worst possible pain) [38].

The REEDA (Redness, Edema, Ecchymosis, Discharge, Approximation) scale: This is a tool used to assess perineal wound healing. It consists of five subscales, each scored from 0 to 3, evaluating redness, edema, ecchymosis, discharge, and wound approximation [39]. The reliability of this tool was examined through multiple evaluations conducted between 6 hours and 10 days after the initial assessment. The reported kappa coefficients, which quantify the level of agreement between assessors, ranged from 0.75 to 0.88 for the discharge metric, 0.46 for edema, 0.42 for ecchymosis, and 0.66 for redness [39].

Adverse Effects and Satisfaction Assessment: An adverse effects checklist was used to document potential treatment-related complications. Maternal satisfaction was assessed on postpartum day 10 using a single-item 5-point Likert scale (“How satisfied are you with your wound healing progress?”), with response options ranging from “Very satisfied” to “Very dissatisfied.” These measures underwent the same validation process as the sociodemographic questionnaire, including independent expert review and cognitive testing to confirm content and face validity.

#### Primary outcome.

- Comparison of the mean scores for wound healing between the group receiving *Nigella sativa* emulgel and the group receiving the placebo, at 10 ± 1 days after the intervention [40,41].

#### Secondary Outcomes.

- Comparison of the mean scores for pain intensity between the group receiving *Nigella sativa* emulgel and the group receiving the placebo, at 1 ± 10 days after the intervention.
- Assessment of the level of satisfaction with the intervention in both the *Nigella sativa* emulgel and placebo groups.
- Determination of adverse events.

### Sample size calculation

The sample size in this study was calculated based on two variables, pain and wound healing, using the G-Power software based on the results of the study by Mohammadi et al. [25]. For the pain variable, considering M1 = 1.2 (intervention group), M2 = 2.6 (control group), SD1 = 1.6, SD2 = 2.1, α = 0.05, and Power = 90%, the sample size was calculated to be 33 participants. Based on the wound healing variable and considering M1 = 1.6 (intervention group), M2 = 3 (control group), SD1 = 1.3, SD2 = 1.6, α = 0.05, and Power = 90%, the sample size was calculated to be 20 participants. Since the determined sample size based on the pain variable was larger, the final sample size was calculated to be 37 participants in each group, considering a 12% dropout rate.

### Statistical analysis

Data were analyzed using SPSS version 26.0 (IBM Corp., Armonk, NY, USA), adhering to intention-to-treat principles. Descriptive statistics are presented as mean ± standard deviation (SD) for normally distributed continuous variables and as frequency (percentage) for categorical variables. The normality of distributions was assessed using multiple complementary methods: evaluation of skewness and kurtosis statistics, and visual inspection of histograms. This analysis revealed non-normal distributions for several REEDA subscale scores (ecchymosis, discharge, and wound approximation).

Primary outcomes (total REEDA scores) were compared between groups using independent samples t-tests at baseline (discharge) and analysis of covariance (ANCOVA) adjusted for baseline scores at follow-up (10 ± 1 days postpartum). For non-normally distributed REEDA subscales, Mann-Whitney U tests were employed.

Secondary outcomes (pain scores) were analyzed following the same analytical approach, with ANCOVA used for follow-up comparisons. Categorical sociodemographic and obstetric variables were compared using Pearson’s χ² tests or Fisher’s exact tests, as appropriate. Ordinal satisfaction data were analyzed using Mann-Whitney U tests. Effect sizes for parametric analyses are reported as mean differences (MD) with 95% confidence intervals. Statistical significance was defined as a two-tailed p-value < 0.05 for all analyses.

## Results

A total of 264 pregnant women were evaluated for eligibility at Taleghani Educational-Therapeutic Hospital, of which 190 were excluded—164 due to ineligibility and 26 due to unwillingness to participate in the study.

**Fig 1:**
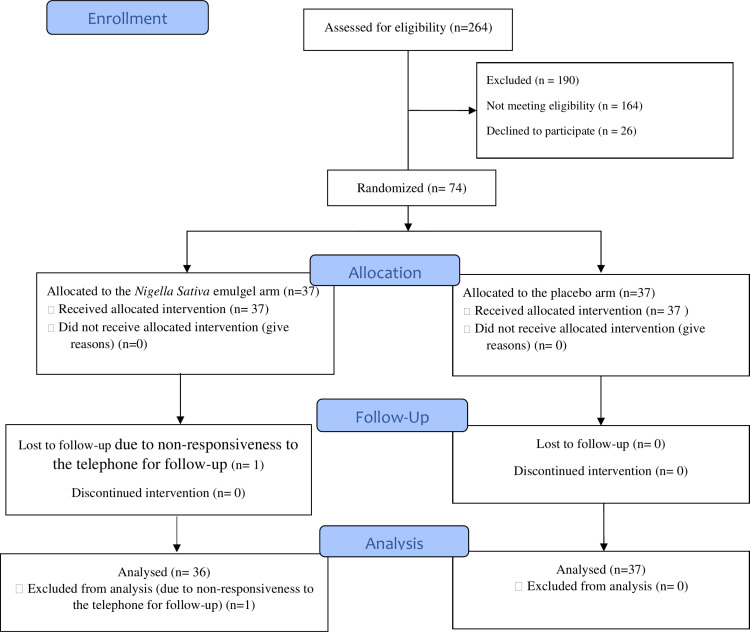
CONSORT flow diagram of the trial.

The follow-up rate was 98.6% at 10 ± 1 days after the intervention. One participant in the *Nigella sativa* emulgel group was lost to follow-up due to unresponsiveness to telephone contact attempts. Consequently, while all 37 participants in each group were included in the baseline analysis (before discharge), the 10 ± 1 days postpartum assessment included 36 participants in the *Nigella Sativa* emulgel group (Fig 1).

The sociodemographic and obstetric characteristics of the women are shown in Table 1. The mean age, BMI, episiotomy incision length, number of skin sutures, durations of the second and third stages of labor, and the baby’s weight were all similar between the two groups, with no statistically significant differences. Similarly, the distribution of participants’ jobs, educational levels, spouses’ jobs and educational levels, family income sufficiency, childbirth agents, and toilet types used at postpartum were comparable between the *Nigella Sativa* emulgel and placebo groups, with no significant differences (p > 0.05).

**Table 1:** Comparison of continuous and categorical variables between *Nigella Sativa* emulgel and placebo groups.

Variable	*Nigella Sativa* Emulgel (n = 37)	Placebo (n = 37)	P-value
**Continuous variables**	**Mean (SD)**	**Mean (SD)**	
Age (years)	28.8 (6.9)	27.5 (7.8)	0.473 ^a^
BMI (kg/m²)	27.1 (3.2)	27.7 (3.9)	0.550 ^a^
Episiotomy incision length (cm)	3.61 (0.90)	3.70 (0.80)	0.754 ^a^
Number of skin sutures	5.37 (1.5)	5.59 (1.4)	0.539 ^a^
Second stage duration (minute)	40.4 (18.2)	44.1 (17.4)	0.383 ^a^
Third stage duration (minute)	13.2 (6.3)	11.7 (4.7)	0.242 ^a^
Baby’s weight (gram)	3123.08 (333.8)	2988.75 (472.8)	0.144 ^a^
**Categorical variables**	**n (%)**	**n (%)**	
**Job**			1.000 ^b^
Housewife	33 (89.2)	33 (89.2)	
Employed	4 (10.8)	4 (10.8)	
**Educational level**			0.586 ^c^
Primary	11 (29.7)	12 (32.4)	
Secondary	8 (21.6)	10 (27.0)	
High school	6 (16.2)	6 (16.2)	
Diploma	9 (24.3)	7 (18.9)	
University	3 (8.1)	2 (5.4)	
**Spouse’s job**			0.216 ^b^
Worker	8 (21.6)	16 (43.2)	
Employee	5 (13.5)	3 (8.1)	
Shopkeeper	7 (18.9)	7 (18.9)	
Self-employed	17 (45.9)	11 (29.7)	
**Spouse’s education level**			0.362 ^c^
Primary	7 (18.9)	11 (29.7)	
Secondary	6 (16.2)	11 (29.7)	
High school	9 (24.3)	6 (16.2)	
Diploma	10 (27.0)	6 (16.2)	
University	5 (13.5)	3 (8.1)	
**Sufficiency of family income**			0.719 ^c^
Completely sufficient	1 (2.7)	0 (0.0)	
Relatively sufficient	33 (89.2)	33 (89.2)	
Insufficient	3 (8.1)	4 (10.8)	
**Childbirth agent**			0.535 ^d^
Midwife	5 (13.5)	8 (21.6)	
Resident	21 (56.8)	21 (56.8)	
Midwifery student with instructor	11 (29.7)	8 (21.6)	
**Toilet types used postpartum**			0.433 ^d^
Squat toilet	25 (67.6)	29 (78.4)	
Sitting toilet	12 (32.4)	8 (21.6)	

^a^ Independent t-tests,

^b^ Fisher’s exact tests,

^c^ Linear-by-Linear Association,

^d^ Pearson Chi-Square.

SD = Standard deviation

### Primary outcome

#### Wound healing.

At 10 ± 1 days postpartum, the *Nigella sativa* emulgel group showed significantly better wound healing than the placebo group, with an adjusted mean difference (AMD) in total REEDA score of −0.79 (95% CI −1.20 to −0.39; p = 0.001) (Table 2).

**Table 2:** Comparison of pain and REEDA scores between *Nigella sativa* emulgel and placebo groups.

Variable	*Nigella sativa* emulgel Mean ± SD	Placebo Mean ± SD	MD (95% CI)	p-value
**Pain score (0–10)**				
Before discharge^†^ (n = 37)	5.16 ± 1.4	5.24 ± 1.7	−0.08 (−0.81 to 0.65)	0.826 ^a^
10 ± 1 days postpartum (n = 36)	0.72 ± 1.2	1.49 ± 1.4	−0.74 (−1.3 to −0.11)	0.021^b^
**REEDA total score (0–15)**				
Before discharge^†^ (n = 37)	2.81 ± 0.93	2.83 ± 0.81	−0.02 (−0.43 to 0.38)	0.913 ^a^
10 ± 1 days postpartum (n = 36)	0.41 ± 0.60	1.20 ± 1.10	−0.79 (−1.20 to −0.39)	0.001 ^b^

^a^ Independent t-test

^b^ Adjusted mean difference (ANCOVA controlling for baseline scores)

† 24 hours post-episiotomy

SD = Standard deviation; CI = Confidence interval; MD = Mean difference

The treatment group exhibited significantly lower scores for three REEDA components compared to placebo: redness (p = 0.037), edema (p = 0.041), and ecchymosis (p = 0.043). No significant differences were found for discharge (p = 0.574) or wound approximation (p = 0.542) (Table 3).

**Table 3:** Comparison of REEDA scale components between *Nigella sativa* emulgel and placebo groups at baseline and follow-up.

REEDA Component	*Nigella sativa* emulgel (Mean ± SD)	Median (Q1, Q3)	Placebo (Mean ± SD)	Median (Q1, Q3)	p-value
**Before discharge** ^†^ ^**(**^**n = 37)**					
Redness (0–3)	1.00 ± 0.66	1.00 (1.00, 1.00)	1.18 ± 0.96	1.00 (0.50, 2.00)	0.331
Edema (0–3)	0.94 ± 0.70	1.00 (0.00, 1.00)	1.00 ± 0.88	1.00 (0.00, 2.00)	0.772
Ecchymosis (0–3)	0.40 ± 0.49	0.00 (0.00, 1.00)	0.29 ± 0.57	0.00 (0.00, 0.50)	0.203
Discharge (0–3)	0.29 ± 0.51	0.00 (0.00, 1.00)	0.11 ± 0.31	0.00 (0.00, 0.00)	0.072
Approximation (0–3)	0.16 ± 0.37	0.00 (0.00, 0.00)	0.24 ± 0.39	0.00 (0.00, 0.00)	0.524
**10 ± 1 days postpartum (n = 36)**					
Redness (0–3)	0.16 ± 0.37	0.00 (0.00, 0.00)	0.43 ± 0.62	0.00 (0.00, 1.00)	0.037
Edema (0–3)	0.11 ± 0.31	0.00 (0.00, 0.00)	0.37 ± 0.63	0.00 (0.00, 1.00)	0.041
Ecchymosis (0–3)	0.05 ± 0.23	0.00 (0.00, 0.00)	0.29 ± 0.66	0.00 (0.00, 0.00)	0.043
Discharge (0–3)	0.02 ± 0.16	0.00 (0.00, 0.00)	0.05 ± 0.22	0.00 (0.00, 0.00)	0.574
Approximation (0–3)	0.05 ± 0.23	0.00 (0.00, 0.00)	0.02 ± 0.16	0.00 (0.00, 0.00)	0.542

a Independent t-test; ^b^ Mann-Whitney U test;

† 24 hours post-episiotomy; SD = Standard deviation; Q1: the first quartile; Q3: the third quartile

### Secondary outcomes

#### Pain intensity.

The *Nigella Sativa* emulgel group showed a significantly lower pain score at 10 ± 1 days after episiotomy compared to the placebo group (AMD: −0.74; 95% CI: −1.3 to −0.11), but the difference was not significant at the time of discharge (MD: −0.8; 95% CI −0.81 to 0.65) (Table 2).

#### Satisfaction with the received intervention.

Satisfaction levels differed significantly between groups (p = 0.046). Participants receiving *Nigella sativa* emulgel reported significantly higher satisfaction, with 32 of 37 (86.5%) rating “very satisfied” or “satisfied” compared to 17 of 30 (56.7%) in the placebo group. Only 4 of 37 (10.8%) intervention participants reported neutral or negative ratings (“equally satisfied and dissatisfied,” “dissatisfied,” or “very dissatisfied”), versus 13 of 30 (43.3%) placebo recipients.

#### Adverse events.

There were no adverse events in participants in either group. Two participants in the placebo group and one participant in the *Nigella Sativa* emulgel group experienced mild itching and redness, which were examined by a physician and treated with antibiotics. These participants were followed up until complete recovery.

## Discussion

The study findings indicate that *Nigella Sativa* emulgel resulted in a significant reduction in pain and improved wound healing from episiotomy within 10 ± 1 days, compared to the placebo group, without any adverse side effects. Furthermore, the level of patient satisfaction with the use of the *Nigella Sativa* emulgel intervention was significantly higher as well.

Wound healing is a complex and dynamic process that involves three main stages: inflammation, proliferation, and remodeling [42]. The inflammatory stage begins immediately after injury and involves blood vessel contraction, coagulation, and the influx of leukocytes such as neutrophils and macrophages to clean up the wound site and initiate the healing process. The proliferative stage focuses on reducing the wound area through contraction and fibroplasia. This stage also involves angiogenesis and re-epithelialization, which are regulated by signaling pathways like Hedgehog [42].

Nigella Sativa, also known as black cumin, has shown great potential in improving wound healing through its diverse biological activities [43–45]. A review of the literature indicates that *Nigella Sativa* can enhance wound healing due to its potent antimicrobial and antioxidant properties [28]. *Nigella Sativa* is rich in bioactive compounds that contribute to its wound healing properties, including high levels of thymoquinone, a major constituent, as well as other volatile monoterpenes such as α-terpinene, α-pinene, sabinene, and 3-carene. These compounds have been shown to exert beneficial effects through enzyme inhibition, cytotoxic activity, and modulation of key inflammatory markers. Additionally, the high antioxidant activity of the non-volatile fraction has also been found to contribute to the wound healing properties of *Nigella Sativa* [45,46].

*Nigella Sativa* has been demonstrated to favorably regulate a range of pro-inflammatory molecules, including cytokines and enzymes, through mechanisms involving the NF-ΚB pathway. These anti-inflammatory effects of *Nigella Sativa* can help mitigate the detrimental impact of excessive inflammation during the wound healing process [47,48].

The study findings indicate that the use of a *Nigella Sativa* emulgel formulation led to a statistically significant reduction in pain levels compared to a placebo control group. Patients in the *Nigella Sativa* group reported a mean pain reduction of 10 ± 1 days, suggesting the treatment was able to effectively manage episiotomy-related pain in a relatively short timeframe. This is an important outcome, as post-episiotomy pain can interfere with activities of daily living, breastfeeding, and the mother’s ability to care for her newborn [49,50].

Furthermore, considering that pharmaceutical interventions can have concerning effects on the concentration of drugs in breast milk and their impact on the newborn, the present intervention with Nigella Sativa, due to its localized action, is likely to have a very minimal effect on the infant. In fact, *Nigella Sativa* has effectively led to increased breastmilk production without any notable side effects [51].

In addition to the analgesic effects, the study also demonstrated that the *Nigella Sativa* emulgel promoted faster wound healing compared to the placebo. Timely and complete wound closure is critical following an episiotomy to prevent complications such as infection, dehiscence, or the development of a fistula [52,53].

In a human clinical study, the aqueous extract of *Nigella Sativa* was observed to facilitate the healing of gingival wounds. This effect was mediated through an increase in the levels of basic fibroblast growth factor (bFGF) and transforming growth factor beta (TGF-β), which are key regulators of wound repair processes. Additionally, the *Nigella Sativa* extract exhibited antioxidant properties, reducing free radical activity, and stimulated the proliferation of gingival fibroblasts [54].

This study found that there were no reported side effects from the use of *Nigella Sativa* among the participants. This aligns with the long history of traditional use of this plant as both a food and a medicine, without any significant adverse effects being documented [55]. Corroborating the findings of the present study, other research on the use of *Nigella Sativa* for wound healing also did not report any adverse side effects [56,57]. This further bolsters the view that *Nigella Sativa* can be used without significant safety risks.

Lastly, the study measured patient satisfaction with the *Nigella Sativa* emulgel, which was found to be significantly higher compared to the placebo group. High patient satisfaction is an important consideration, as it suggests the treatment was acceptable, convenient, and met the needs and expectations of the end-users [58].

### Strength and limitation

The study employed a randomized, triple-blind controlled design to reduce bias and enhance the validity of its findings. The implementation of clear eligibility criteria and standardized treatment protocols allowed for direct comparisons between the *Nigella Sativa* emulgel and placebo. Furthermore, the use of multiple assessment tools facilitated a comprehensive evaluation of outcomes.

Conversely, the study’s single-center design limited the generalizability of the results. The 10-day follow-up period did not adequately capture long-term effects. Additionally, the subjective nature of assessment tools, such as the VAS, impacted the reliability of the findings, underscoring the necessity for further research.

### Implications for research

More comprehensive studies with longer-term follow-up are needed to thoroughly evaluate the potential for recurrence of perineal trauma or long-term impacts on pelvic floor function. This would provide more definitive conclusions about the efficacy and safety of *Nigella Sativa* emulgel for episiotomy management. Additionally, comparative effectiveness research pitting the *Nigella Sativa* emulgel against standard episiotomy care or other emerging treatment options could help elucidate its relative merits and optimal positioning within the clinical management of perineal injuries.

### Implications for practice

The positive impacts on wound healing and pain observed in this study, combined with the relative ease of application and lack of significant safety concerns, indicate that *Nigella Sativa* emulgel may be a valuable addition to the clinical toolkit for obstetricians and midwives caring for women who have undergone episiotomy procedures. However, further research is needed to fully establish the efficacy, safety, and optimal implementation protocols before this intervention can be confidently recommended for widespread clinical adoption.

## Conclusion

*Nigella sativa* emulgel is a promising natural therapeutic option for managing pain and promoting wound healing following episiotomy procedures. The treatment was found to be effective, safe, and well-received by patients. These findings warrant further investigation through larger, multi-center clinical trials to confirm the reproducibility and generalizability of the results.

## Supporting information

S1 Dataset Supplementary data. (XLSX)

S1 File Protocol for a triple-blind randomized controlled trial on the efficacy of *Nigella Sativa* emulgel in episiotomy wound healing and pain reduction in primiparous women. (DOCX)

S1 Checklist CONSORT 2010 checklist of information to include when reporting a randomised trial. (DOCX)
