# Supplementary material for: The effect of Nigella Sativa emulgel on episiotomy wound healing and pain intensity in primiparous women: A triple-blind randomized controlled trial
Source: PLoS One. 2025 Jun 4;20(6):e0325112. doi: 10.1371/journal.pone.0325112 (PMC12136343; doi:10.1371/journal.pone.0325112)
Supplement: S1 File — (DOCX) [file pone.0325112.s002.docx]

**Protocol for a Triple-Blind Randomized Controlled Trial on the Efficacy of *Nigella Sativa* Emulgel in Episiotomy Wound Healing and Pain Reduction in Primiparous Women**

**Background**

Episiotomy, a surgical incision of the perineum during the second stage of labor, will remain one of the most common obstetric procedures worldwide, particularly among primiparous women [1]. Despite recommendations from the World Health Organization (WHO) and the International Federation of Gynecology and Obstetrics (FIGO) to restrict its use, episiotomy rates will continue to be disproportionately high in many regions, reaching up to 87% in primiparous women in Iran [2,3].

Post-episiotomy complications, including persistent pain, infection, and delayed wound healing, will significantly affect maternal postpartum recovery [4]. Chronic perineal pain following episiotomy will be reported in up to 20% of women, with some experiencing discomfort for several months postpartum [5]. This pain may interfere with maternal-infant bonding, breastfeeding, and daily activities, while also increasing the risk of postpartum depression [6,7].

Current pharmacological interventions for episiotomy-related pain, such as NSAIDs and opioids, will carry risks of systemic side effects, including neonatal exposure through breast milk [8,9]. Non-pharmacological approaches, including cold therapy, laser treatment, and herbal remedies (e.g., lavender and aloe vera), will show promise but will require further validation [10,11].

*Nigella sativa* (black seed) will be extensively studied for its anti-inflammatory, antimicrobial, and wound-healing properties [12]. Its active constituent, thymoquinone, will modulate key inflammatory pathways (NF-κB) and enhance fibroblast proliferation, accelerating tissue repair [13]. Additionally, *Nigella sativa* oil will demonstrate efficacy in dermatological applications, improving wound closure and reducing oxidative stress [14]. Given its FDA classification as generally recognized as safe (GRAS) for dietary use [15], *Nigella sativa* emulgel will present a compelling natural alternative for episiotomy management.

This study will rigorously evaluate the efficacy of *Nigella sativa* emulgel in improving episiotomy wound healing and reducing pain intensity in primiparous women, compared to a placebo.

**Methods**

**Study Design**

A triple-blind (participants, outcome assessors, data analysts), randomized, placebo-controlled, parallel-group trial will be conducted at Taleghani Hospital, a tertiary care center in Tabriz, Iran. The study will adhere to CONSORT guidelines for clinical trials.

**Dates of Recruitment**

Recruitment period: May 19, 2023 – April 15, 2024

Follow-up duration: 10 ± 1 days postpartum (to assess short-term healing and pain resolution)

**Participant Characteristics**

Population: Healthy primiparous women (first vaginal delivery)

Sample size: 74 participants (37 per group), calculated via G*Power based on wound healing and pain outcomes from prior studies [16].

**Inclusion criteria**

Age ≥18 years

Singleton pregnancy

Mediolateral episiotomy performed by a trained clinician

Willingness to adhere to the study protocol

**Exclusion Criteria**

Medical comorbidities (e.g., diabetes, immunosuppression, collagen disorders) that may impair wound healing

Perineal trauma beyond standard episiotomy (third-/fourth-degree tears)

Allergy to *Nigella sativa* or emulgel components

Postpartum hemorrhage

Operative delivery (forceps/vacuum assistance)

**Randomization & Blinding**

Randomization: Computer-generated block randomization (blocks of 4 and 6) via Random.org

Allocation concealment: Sequentially numbered, opaque containers will be dispensed by a third-party pharmacist.

**Blinding**

Participants will receive identical-appearing emulgel.

Outcome assessors will be blinded to group allocation.

Statisticians will analyze coded data.

**Interventions**

*Nigella Sativa* Emulgel Group

Formulation:

Oil phase: 5% *Nigella sativa* oil (cold-pressed, standardized to 0.5% thymoquinone).

Aqueous phase: 2% carbomer 940 gel, preservatives (methyl/propyl paraben).

Emulsifiers: Span 80 (0.5%) and Tween 80 (0.5%).

**Application protocol**

Will be applied topically to the episiotomy site 3 times daily for 7 days post-discharge.

Participants will record adherence via a diary.

Placebo Group

Formulation: Identical emulgel base (paraffin oil replacing *Nigella sativa* oil).

Application: Same regimen as the intervention group.

**Outcome Measures**

Primary Outcome

Wound healing will be assessed via the REEDA scale (Redness, Edema, Ecchymosis, Discharge, Approximation) at 10 ± 1 days postpartum [17].

Secondary Outcomes

Pain intensity will be measured by Visual Analog Scale (VAS, 0–10) at discharge and follow-up [18].

Patient satisfaction (5-point Likert scale: "Very satisfied" to "Very dissatisfied").

Adverse events (e.g., itching, infection, allergic reactions).

**Data Collection & Analysis**

Data Collection Tools

Baseline questionnaire: Demographics, obstetric history, episiotomy length, suture count.

REEDA Scale: Validated for perineal healing (κ = 0.42–0.88 for subscales) [17].

VAS Pain Scale: Standardized 10-cm ruler.

**Statistical Analysis**

Software: SPSS v26 (IBM Corp.).

Primary analysis: ANCOVA (adjusted for baseline REEDA scores).

Secondary analyses:

Independent t-tests for normally distributed continuous data.

Mann-Whitney U test for non-parametric data (e.g., REEDA subscales).

Chi-square/Fisher’s exact test for categorical variables.

Effect size reporting: Mean difference (MD) with 95% CI.

**References**

1. Ghulmiyyah L, Sinno S, Mirza F, Finianos E, Nassar AH. Episiotomy: history, present and future - a review. J Matern Fetal Neonatal Med. 2022;35(7):1386-1391. doi: 10.1080/14767058.2020.1755647.
2. Rasouli M, Keramat A, Khosravi A, Mohabatpour Z. Prevalence and factors associated with episiotomy in Shahroud City, northeast of Iran. Int J Womens Health Reprod Sci 2016, 4(3):125-129.
3. Mousavi SHS, Miri M, Farzaneh F. Episiotomy and its complications. Zahedan J Res Med Sci. 2020, 23, 104127.
4. Gün İ, Doğan B, Özdamar Ö. Long- and short-term complications of episiotomy. Turk J Obstet Gynecol. 2016;13(3):144-148. doi: 10.4274/tjod.00087.
5. Turmo M, Echevarria M, Rubio P, Almeida C. Development of chronic pain after episiotomy. Rev Esp Anestesiol Reanim. 2015;62(8):436-42. English, Spanish. doi: 10.1016/j.redar.2014.10.008.
6. Eisenach JC, Pan PH, Smiley R, Lavand'homme P, Landau R, Houle TT. Severity of acute pain after childbirth, but not type of delivery, predicts persistent pain and postpartum depression. Pain. 2008;140(1):87-94. doi: 10.1016/j.pain.2008.07.011.
7. Lu L, Shen Y. Postpartum pain and the risk of postpartum depression: A meta-analysis of observational studies. J Obstet Gynaecol Res. 2024;50(3):358-365. doi: 10.1111/jog.15850.
8. East CE, Sherburn M, Nagle C, Said J, Forster D. Perineal pain following childbirth: prevalence, effects on postnatal recovery and analgesia usage. Midwifery. 2012;28(1):93-7. doi: 10.1016/j.midw.2010.11.009.
9. Luxey X, Lemoine A, Dewinter G, Joshi GP, Le Ray C, Raeder J, et al; PROSPECT Working Group of the European Society of Regional Anesthesia and Pain Therapy. Acute pain management after vaginal delivery with perineal tears or episiotomy. Reg Anesth Pain Med. 2024: rapm-2024-105478. doi: 10.1136/rapm-2024-105478.
10. Hables RM. Effect of olive oil, lavender oil and placebo on pain intensity and healing of episiotomy in women. Tnta Sci Nurs J. 2021; 20:47-63.
11. Chellappan K. A systemic review on impact of aloe vera emulgel in episiotomy pain and wound healing. J ReAttach Therapy Dev Diversit. 2023;6(9s):1493-500.
12. Hannan MA, Rahman MA, Sohag AAM, Uddin MJ, Dash R, Sikder MH, et al. Black Cumin (Nigella sativa L.): A Comprehensive Review on Phytochemistry, Health Benefits, Molecular Pharmacology, and Safety. Nutrients. 2021;13(6):1784. doi: 10.3390/nu13061784.
13. Rashwan HK, Mahgoub S, Abuelezz NZ, Amin HK. Black Cumin Seed (Nigella sativa) in inflammatory disorders: Therapeutic potential and promising molecular mechanisms. Drugs Drug Candid. 2023; 2(2):516-537.
14. Almuhayawi M. Efficacy of Nigella sativa in Wound Healing. Indian Journal of Pharmaceutical Sciences 2023: 85:173-181.
15. Burdock GA. Assessment of black cumin (Nigella sativa L.) as a food ingredient and putative therapeutic agent. Regul Toxicol Pharmacol. 2022; 128:105088. doi: 10.1016/j.yrtph.2021.105088.
16. Mohammadi A, Mohammad-Alizadeh-Charandabi S, Mirghafourvand M, Javadzadeh Y, Fardiazar Z, Effati-Daryani F. Effects of cinnamon on perineal pain and healing of episiotomy: a randomized placebo-controlled trial. J Integr Med. 2014;12(4):359-66. doi: 10.1016/S2095-4964(14)60025-X.
17. Alvarenga MB, Francisco AA, de Oliveira SM, da Silva FM, Shimoda GT, Damiani LP. Episiotomy healing assessment: Redness, Oedema, Ecchymosis, Discharge, Approximation (REEDA) scale reliability. Rev Lat Am Enfermagem. 2015;23(1):162-8. doi: 10.1590/0104-1169.3633.2538.
18. Crichton N. Visual analogue scale (VAS). J Clin Nurs. 2001;10(5):706.
