# Supplementary material for: The effect of Nigella Sativa emulgel on episiotomy wound healing and pain intensity in primiparous women: A triple-blind randomized controlled trial
Source: PLoS One. 2025 Jun 4;20(6):e0325112. doi: 10.1371/journal.pone.0325112 (PMC12136343; doi:10.1371/journal.pone.0325112)
Supplement: S1 Checklist — (DOCX) [file pone.0325112.s003.docx]

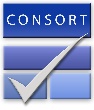
CONSORT 2010 checklist of information to include when reporting a randomised trial*

| Section/Topic | Item No | Checklist item | Reported on page No |
| --- | --- | --- | --- |
| Title and abstract | | | |
|  | 1a | Identification as a randomised trial in the title | **Page 1** (Title: "The effect of Nigella Sativa emulgel on pain and healing following episiotomy wound in primiparous women: a triple-blind randomized controlled trial") |
|  | 1b | Structured summary of trial design, methods, results, and conclusions (for specific guidance see CONSORT for abstracts) | **Page 2** (Abstract) |
| Introduction | | | |
| Background and objectives | 2a | Scientific background and explanation of rationale | **Page 3-5** (Introduction) |
|  | 2b | Specific objectives or hypotheses | **Page 5** (Introduction) |
| Methods | | | |
| Trial design | 3a | Description of trial design (such as parallel, factorial) including allocation ratio | **Page 4** (Methods: "randomized, triple-blind controlled clinical trial") |
|  | 3b | Important changes to methods after trial commencement (such as eligibility criteria), with reasons | **Not reported** (No changes were done) |
| Participants | 4a | Eligibility criteria for participants | **Page 5** (Methods: "Eligibility criteria") |
|  | 4b | Settings and locations where the data were collected | **Page 5** (Methods: "Taleghani Educational-Medical Center in Tabriz, Iran") |
| Interventions | 5 | The interventions for each group with sufficient details to allow replication, including how and when they were actually administered | **Page 6** (Methods: "Interventions and follow-up") |
| Outcomes | 6a | Completely defined pre-specified primary and secondary outcome measures, including how and when they were assessed | **Page 9-10** (Methods: "Primary Outcome" and "Secondary Outcomes") |
|  | 6b | Any changes to trial outcomes after the trial commenced, with reasons | Not reported (No changes done) |
| Sample size | 7a | How sample size was determined | **Page 10** (Methods: "Sample size calculation") |
|  | 7b | When applicable, explanation of any interim analyses and stopping guidelines | N/A (No interim analyses done) |
| Randomisation: |  |  |  |
| Sequence generation | 8a | Method used to generate the random allocation sequence | **Page 5** (Methods: "Randomization and blinding") |
|  | 8b | Type of randomisation; details of any restriction (such as blocking and block size) | **Page 6** (Methods: "block randomization with variable block sizes of 4 and 6") |
| Allocation concealment mechanism | 9 | Mechanism used to implement the random allocation sequence (such as sequentially numbered containers), describing any steps taken to conceal the sequence until interventions were assigned | **Page 6** (Methods: "identical, opaque, and sequentially numbered containers") |
| Implementation | 10 | Who generated the random allocation sequence, who enrolled participants, and who assigned participants to interventions | **Page 6** (Methods: "generation of the treatment allocation sequence and the preparation of the containers were conducted by an individual who was not involved in the participant enrollment, treatment allocation, or data collection processes") |
| Blinding | 11a | If done, who was blinded after assignment to interventions (for example, participants, care providers, those assessing outcomes) and how | **Page 5** (Methods: "triple-blind (participants, outcome assessors, and data analysts)") |
|  | 11b | If relevant, description of the similarity of interventions | **Page 7-8** (Methods: "Preparation of Nigella Sativa and Placebo Emulgels") |
| Statistical methods | 12a | Statistical methods used to compare groups for primary and secondary outcomes | **Page 10-11** (Methods: "Statistical Analysis") |
|  | 12b | Methods for additional analyses, such as subgroup analyses and adjusted analyses | **Page 10-11** (Methods: "ANCOVA tests, adjusted for baseline scores") |
| Results | | | |
| Participant flow (a diagram is strongly recommended) | 13a | For each group, the numbers of participants who were randomly assigned, received intended treatment, and were analysed for the primary outcome | **Page 11** and figure 1 (Results: "Lost to follow-up due to non-responsiveness to the telephone for follow-up") |
|  | 13b | For each group, losses and exclusions after randomisation, together with reasons | **Page 11** and figure 1 (Results: "Lost to follow-up due to non-responsiveness to the telephone for follow-up") |
| Recruitment | 14a | Dates defining the periods of recruitment and follow-up | **Page 5** (Methods: "from May 19, 2023 to April 15, 2024") |
|  | 14b | Why the trial ended or was stopped | N/A (Trial ended as planned) |
| Baseline data | 15 | A table showing baseline demographic and clinical characteristics for each group | **Page 28** (Table 1) |
| Numbers analysed | 16 | For each group, number of participants (denominator) included in each analysis and whether the analysis was by original assigned groups | **Page 11** (Results: "Analysed (n=36)") and **figure 1** |
| Outcomes and estimation | 17a | For each primary and secondary outcome, results for each group, and the estimated effect size and its precision (such as 95% confidence interval) | **Page 12 and Page 29** (Table 2) |
|  | 17b | For binary outcomes, presentation of both absolute and relative effect sizes is recommended | **Not applicable** |
| Ancillary analyses | 18 | Results of any other analyses performed, including subgroup analyses and adjusted analyses, distinguishing pre-specified from exploratory | **Page 12 (results: "ANCOVA tests, adjusted for baseline scores")** |
| Harms | 19 | All important harms or unintended effects in each group (for specific guidance see CONSORT for harms) | **Page 12** (Results: "Adverse Events") |
| Discussion | | | |
| Limitations | 20 | Trial limitations, addressing sources of potential bias, imprecision, and, if relevant, multiplicity of analyses | **Page 15-16** (Discussion: "Strength and limitation") |
| Generalisability | 21 | Generalisability (external validity, applicability) of the trial findings | **Page 15-16** (Discussion: "Strength and limitation") |
| Interpretation | 22 | Interpretation consistent with results, balancing benefits and harms, and considering other relevant evidence | **Page 13-15** (Discussion) |
| Other information | | |  |
| Registration | 23 | Registration number and name of trial registry | **Page 6** (Methods: "registered the study in the Iranian Registry of Clinical Trials (IRCT20120718010324N68)" |
| Protocol | 24 | Where the full trial protocol can be accessed, if available | N/A |
| Funding | 25 | Sources of funding and other support (such as supply of drugs), role of funders | **Page 18** (Funding: "The study was funded by Tabriz University of Medical Sciences") |

Citation: Schulz KF, Altman DG, Moher D, for the CONSORT Group. CONSORT 2010 Statement: updated guidelines for reporting parallel group randomised trials. BMC Medicine. 2010;8:18.
© 2010 Schulz et al. This is an Open Access article distributed under the terms of the Creative Commons Attribution License (<http://creativecommons.org/licenses/by/2.0>), which permits unrestricted use, distribution, and reproduction in any medium, provided the original work is properly cited.

*We strongly recommend reading this statement in conjunction with the CONSORT 2010 Explanation and Elaboration for important clarifications on all the items. If relevant, we also recommend reading CONSORT extensions for cluster randomised trials, non-inferiority and equivalence trials, non-pharmacological treatments, herbal interventions, and pragmatic trials. Additional extensions are forthcoming: for those and for up-to-date references relevant to this checklist, see [www.consort-statement.org](http://www.consort-statement.org).
